# *Isocitrate dehydrogenase 1*-mutated cancers are sensitive to the green tea polyphenol epigallocatechin-3-gallate

**DOI:** 10.1186/s40170-019-0198-7

**Published:** 2019-05-20

**Authors:** Tom H. Peeters, Krissie Lenting, Vincent Breukels, Sanne A. M. van Lith, Corina N. A. M. van den Heuvel, Remco Molenaar, Arno van Rooij, Ron Wevers, Paul N. Span, Arend Heerschap, William P. J. Leenders

**Affiliations:** 1 Department of Radiology and Nuclear Medicine, Radboud university medical center, PO Box 9101, 6500 Nijmegen, HB The Netherlands; 2 Department of Biochemistry, Radboud Institute for Molecular Life Sciences, Geert Grooteplein 26, 6525 Nijmegen, GA The Netherlands; 3 Department of Medical Biology, Cancer Center Amsterdam at the Academic Medical Center, Meibergdreef 15, 1105 Amsterdam, AZ The Netherlands; 4 Department of Laboratory Medicine, Radboud university medical center, PO Box 9101, 6500 Nijmegen, HB The Netherlands; 5 Department of Radiation Oncology, Radiotherapy and OncoImmunology Laboratory, Radboud university medical center, PO Box 9101, 6500 Nijmegen, HB The Netherlands

**Keywords:** IDH mutations, Metabolism, EGCG, Radiotherapy, Glutamate

## Abstract

**Background:**

Mutations in isocitrate dehydrogenase 1 (*IDH1*) occur in various types of cancer and induce metabolic alterations resulting from the neomorphic activity that causes production of *D*-2-hydroxyglutarate (*D-*2-HG) at the expense of α-ketoglutarate (α-KG) and NADPH. To overcome metabolic stress induced by these alterations, *IDH*-mutated (*IDH*^*mut*^) cancers utilize rescue mechanisms comprising pathways in which glutaminase and glutamate dehydrogenase (GLUD) are involved. We hypothesized that inhibition of glutamate processing with the pleiotropic GLUD-inhibitor epigallocatechin-3-gallate (EGCG) would not only hamper *D-*2-HG production, but also decrease NAD(P)H and α-KG synthesis in *IDH*^*mut*^ cancers, resulting in increased metabolic stress and increased sensitivity to radiotherapy.

**Methods:**

We performed ^13^C-tracing studies to show that HCT116 colorectal cancer cells with an *IDH1*^*R132H*^ knock-in allele depend more on glutaminolysis than on glycolysis for the production of *D*-2-HG. We treated HCT116 cells, HCT116-*IDH1*^*R132H*^ cells, and HT1080 cells (carrying an *IDH1*^*R132C*^ mutation) with EGCG and evaluated *D-*2-HG production, cell proliferation rates, and sensitivity to radiotherapy.

**Results:**

Significant amounts of ^13^C from glutamate accumulate in *D-*2-HG in HCT116-*IDH1*^*wt/R132H*^ but not in HCT116-*IDH1*^*wt/wt*^. Preventing glutamate processing in HCT116-*IDH1*^*wt/R132H*^ cells with EGCG resulted in reduction of *D-*2-HG production. In addition, EGCG treatment decreased proliferation rates of *IDH1*^*mut*^ cells and at high doses sensitized cancer cells to ionizing radiation. Effects of EGCG in IDH-mutated cell lines were diminished by treatment with the IDH1^mut^ inhibitor AGI-5198.

**Conclusions:**

This work shows that glutamate can be directly processed into *D-*2-HG and that reduction of glutamatolysis may be an effective and promising new treatment option for *IDH*^*mut*^ cancers.

**Electronic supplementary material:**

The online version of this article (10.1186/s40170-019-0198-7) contains supplementary material, which is available to authorized users.

## Background

Acquisition of hotspot mutations in *IDH1* and *IDH2* are key events in the development of various types of cancer. The mutations are found in 80–90% of gliomas [1–3], in substantial percentages of acute myeloid leukemia [4], chondrosarcoma [5], osteosarcoma [6], and intrahepatic cholangiocarcinoma [7], and are sporadically found in other cancer types [8, 9].

IDH1 and IDH2 are NADP^+^-dependent homodimeric enzymes that oxidize isocitrate (ICT) to α-ketoglutarate (α-KG) in cytosol and mitochondria, respectively [10]. The NADPH produced by these reactions contributes to the reductive potential of the cell [11]. Cancer-related mutations in *IDH1* and *IDH2* are mostly heterozygous and are always hotspot mutations involving arginine residues R132 in IDH1 and R140 or R172 in IDH2. The mutated subunits have acquired a neomorphic activity of reducing α-KG to *D*-2-hydroxyglutarate (*D-*2-HG) while oxidizing NAPDH [12, 13]. Accumulation of *D-*2-HG competitively inhibits α-KG-dependent enzymes, including the ten-eleven translocation (TET) family of methylcytosine dioxygenases, resulting in a CpG island hypermethylator phenotype that is considered as a first step in malignant transformation [14, 15]. Whereas *IDH* mutations are involved in the initial steps of carcinogenesis, the metabolic and oxidative stress that comes with the mutation may eventually slow down tumor progression, explaining the better survival of patients carrying *IDH*^*mut*^ gliomas [16, 17]. *IDH* mutations are however not associated with prolonged survival in non-glioma cancer patients, indicating tissue-specific effects that are currently not understood [1, 3, 18].

Small molecule inhibitors of mutant IDH1 and IDH2 enzymes have been developed to prevent the production of the alleged oncometabolite *D-*2-HG [19, 20]. However, blocking *D-*2-HG production also blocks NADPH oxidation and consequently decreases oxidative stress, desensitizing *IDH*^*mut*^ cells for radiotherapy, chemotherapy, and inhibitors of poly-ADP ribose polymerase (PARP), an important enzyme involved in DNA double-strand break (DSB) repair [21–24].

To improve the clinical outcomes of patients with *IDH*^*mut*^ cancers, it is essential to increase, rather than decrease, metabolic stress. We previously showed that clinical *IDH*^*mut*^ gliomas have dramatically altered expression profiles of genes involved in metabolism as compared to *IDH*^*wt*^ gliomas. Based on these data, we proposed a model in which *IDH*^*mut*^ gliomas utilize the neurotransmitter glutamate and lactate as fuels [25], whereas *IDH*^*wt*^ gliomas predominantly use glucose [26]. According to that model, the shortage of α-KG in *IDH*^*mut*^ gliomas is partially rescued by direct import of glutamate that is converted to α-KG by the NAD^+^-/NADP^+^-dependent enzymes glutamate dehydrogenase 1/2 (GLUD1/2). In *IDH*^*mut*^ non-gliomas, residing in environments with low glutamate concentrations, this rescue pathway may start with the import of glutamine that is first converted to glutamate by mitochondrial glutaminase (GLS), followed by GLUD1/2-mediated further processing to α-KG. Multiple studies have shown that glutamine is a major carbon donor for *D*-2-HG [27–29]. Alpha-KG can then be shuttled into the TCA cycle or converted to *D-*2-HG. We therefore hypothesized that inhibition of glutamate processing in *IDH*^*mut*^ cancer cells would not only prevent *D-*2-HG production, but also NAD(P) H and α-KG synthesis, thus increasing metabolic stress and sensitizing *IDH*^*mut*^ cancers to radiotherapy and chemotherapy [25, 30].

To test how different nutrients contribute to *D*-2-HG production in non-glioma tumors, we here employed HCT116 and HCT116-*IDH1*^*wt/R132H*^ knock-in colorectal cancer cells and HT1080 cells, a fibrosarcoma cell line containing an endogenous IDH1^R132C^ mutation. We performed carbon tracing studies and investigated the effects of epigallocatechin-3-gallate (EGCG), an inhibitor of GLUD1/2 and of NADP-dependent enzymes, on *D-*2-HG synthesis and radiosensitivity of these cell lines.

## Methods

### Cell lines and compounds

HCT116-*IH1*^*wt/wt*^ (parental) and HCT116-*IDH1*^*wt/R132H*^ knock-in human colorectal cell lines were generated by AAV targeting technology GENESIS [31] and obtained from Horizon Discovery (Cambridge, UK). HT1080 fibrosarcoma cells (containing an endogenous *IDH1*^*wt/R132C*^ mutation) were a kind gift of Dr. W. Hendriks (Dept. of Cell Biology, Radboudumc). Cell lines were cultured in DMEM (LONZA, Basel, Switzerland) supplemented with 10% FCS (Gibco, Waltham, MA) and 40 μg/μl gentamycin (Centrafarm, Etten-Leur, the Netherlands). Cell lines were checked for IDH1^R132H^ expression by Western blotting of cytosolic protein extracts, using a mutation-specific antibody (Dianova, Hamburg, Germany; DIAH09). All experiments in this study were performed with cells below passage number 25 as IDH1^R132H^ expression levels gradually dropped at higher passage numbers (data not shown). All chemicals were obtained from Sigma Aldrich (St. Louis, MO) unless stated otherwise. EGCG (E4268) was stored in DMSO at a concentration of 25 mM under nitrogen gas and kept from light, or dissolved in distilled water directly before use. The IDH1^mut^ inhibitor AGI-5198 was from MedChemExpress (Monmouth Junction, NJ).

### ^13^C-isotope tracing experiments

**Fig. 1:**
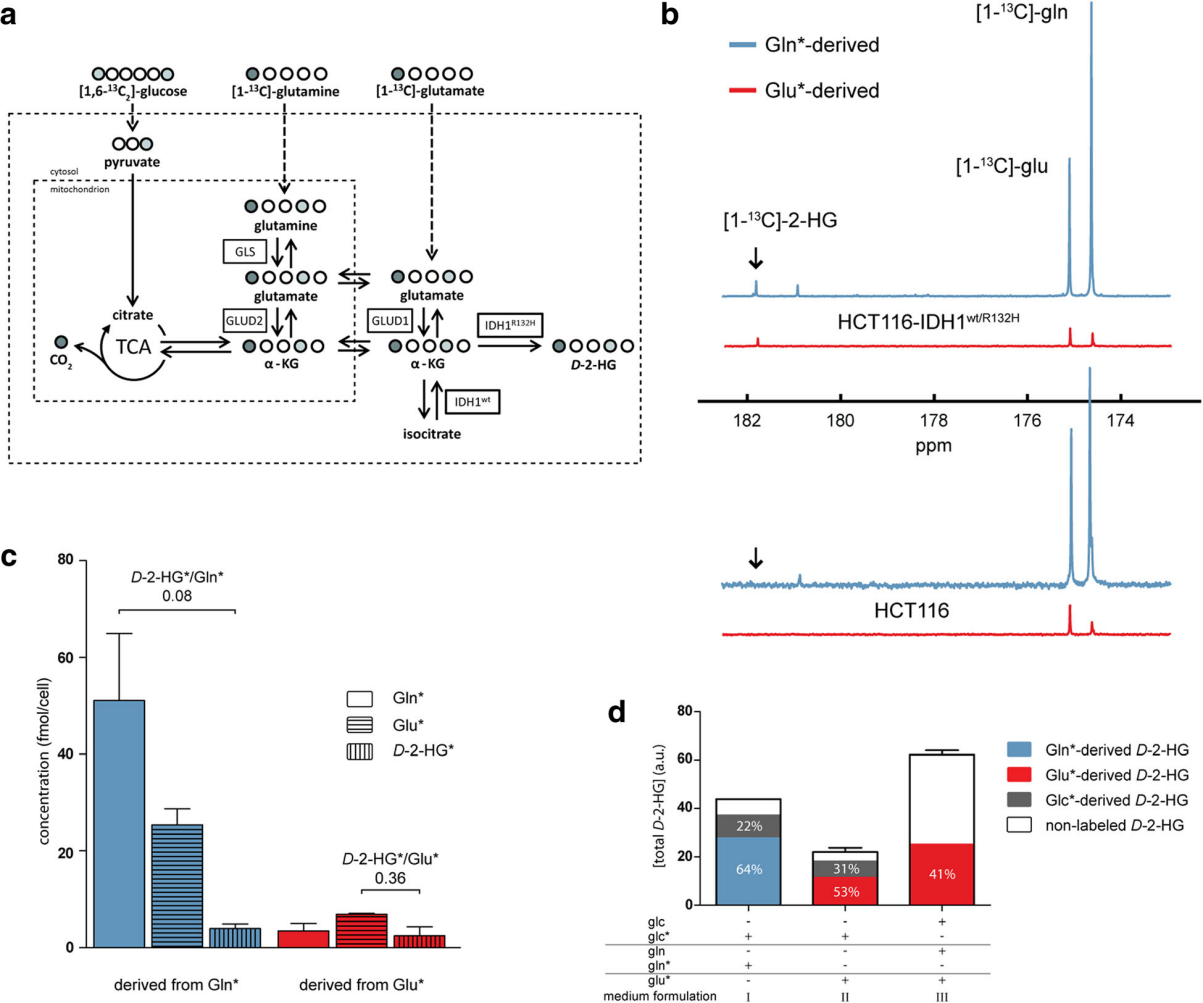
Carbon sources for *D-*2-HG production and ^13^C tracing studies. **a** Schematic model of metabolic pathways involved in the production of *D-*2-HG from extracellular glucose, glutamine, and glutamate; the ^13^C labeling patterns following incubation in culture medium containing ^13^C-labeled glucose (light circles) and glutamine or glutamate (dark circles). **b** Example of ^13^C NMR spectra of extracts of HCT116-*IDH1*^*wt/R132H*^ and HCT116 cells cultured in medium with [1-^13^C]-glutamine (Gln*, blue) or [1-^13^C]-glutamate (Glu*, red). [1-^13^C]-*D-*2-HG (*D-*2-HG*) was detected at 181.8 ppm and could not be observed in parental HCT116 cells. **c** Quantified NMR results showing levels of Glu*, Gln*, and *D-*2-HG* in HCT116-*IDH1*^*wt/R132H*^ cells, after incubation in medium with Gln* (blue) or Glu* (red). **d** LC-MS analysis of *D-*2-HG pool fractional enrichments in HCT116-*IDH1*^*wt/R132H*^ cells after incubation with ^13^C-labeled substrates. Bar I displays the total amount of *D-*2-HG and the fractions that were derived from Gln* (blue) and Glc* (gray). Results were obtained from two separate measurements: in the first measurement cells were incubated in DMEM with Glc and Gln*; in the second measurement DMEM was supplemented with Glc* and Gln*. The difference in labeled fractions from these two measurements was assigned as the Glc* fraction. In a parallel experiment Gln and Gln* were substituted by Glu and Glu* respectively. Bar II shows the total amount of *D-*2-HG and the fractions that were derived from Glu* (red) and Glc* (gray). A third experiment was performed with DMEM containing Glu*, Gln, and Glc. Bar III displays the total amount of *D-*2-HG and the fraction that was derived from Glu* (red). Provided that both Glu and Gln are available from the culture medium, production of Glu-derived *D-*2-HG (red) was substantial. Gln* and Glc* fractions were not measured in experiment III. Supplemented metabolite concentrations were always the same: 5.5 mM glucose and 4 mM glutamine and/or 4 mM glutamate

Nuclear magnetic resonance (NMR) spectroscopy and LC-MS experiments were performed to investigate the contribution of glutamine (Gln), glutamate (Glu), and glucose (Glc) as carbon donors for *D-*2-HG (see Fig. 1a). For NMR, HCT116-*IDH1*^*wt/R132H*^ and HCT116 cells were grown to 50% confluency in T175 culture flasks (Greiner Bio-One, Kremsmünster, Austria) and incubated with glutamine-free DMEM (Gibco) supplemented with 10% FCS and 4 mM [1-^13^C]-glutamine or 4 mM [1-^13^C]-glutamate. EGCG (100 μM final concentration in distilled H_2_O) or solvent control was administered 2 h prior to the start of incubation. After 20 h of incubation, cells were placed on ice, washed twice with ice-cold PBS, and lysed in 2.5 ml methanol (MeOH) (− 20 °C) containing 280 mM formic acid as a ^1^H and ^13^C (naturally abundant) NMR reference compound. After 10 min, cell material was collected with a rubber policeman, thoroughly vortexed and centrifuged for 5 min at 1200×*g* to precipitate proteins. The protein content of the precipitated pellets was measured using a Pierce BCA protein assay kit (ThermoScientific, Rockford, IL) and used for data normalization. The metabolites in the supernatant were dried in a SpeedVac evaporator (Savant, Waltham, MA) and redissolved in 400 μl D_2_O for NMR analysis (Avance III 500 MHz, Bruker BioSpin, Rheinstetten, Germany).

NMR spectra were acquired with pulse-acquire experiments. For ^1^H NMR, the settings were TR = 18 s, 90° flip angle, NS = 16; and for ^13^C, TR = 5.1 s, 30° flip angle, NS = 7000, and proton-decoupling. The spectra were analyzed with Bruker Topspin software. Integrated peak intensities of *D-*2-HG, Glu, and Gln were corrected for *T*_1_ saturation, number of contributing spins, and cell number and were referenced to formic acid to obtain concentrations.

Because total amounts of *D-*2-HG are difficult to obtain from ^1^H spectra, due to overlapping resonances of *D*-2-HG and glutamate, total and ^13^C-labeled *D-*2-HG pools were examined with LC-MS. To this end, HCT116 and HCT116-*IDH1*^*wt/R132H*^ cells were grown to 50% confluency in 10 cm culture dishes (Greiner Bio-One) and incubated with Glc- and Gln-free DMEM with and without 10% FCS and different combinations of non-labeled and ^13^C-labeled Glc, Gln, or Glu. In all experiments, the final total Glc concentration was 5.5 mM and Glu and/or Gln concentrations were 4 mM. LC-MS was performed as described before [32].

### Proliferation assays

Cells were seeded in 96-well plates at 500 cells/well (HCT116 cell lines) or 1000 cells/well (HT1080) and left to adhere overnight. The following day varying concentrations of EGCG or vehicle were added. For AGI-5198 experiments, cells were cultured at least 3 days in the presence of 5 μM AGI-5198, and the compound was left on the cells during the entire experiment [21]. At days 2, 4, and 6 after seeding, total cell protein content was measured using SRB assays, as described [33]. In short, cells were washed twice with PBS and fixated overnight at 4 °C in 10% (*w*/*v*) trichloroacetic acid. After fixation, plates were washed four times with distilled H_2_O and stored at − 20 °C until analysis. Plates were stained with 0.5% (*w*/*v*) SRB dissolved in 1% (*v*/*v*) acetic acid (Merck, Darmstadt, Germany) and incubated in the dark for 20 min. After washing four times with 1% acetic acid, plates were dried at 60 °C. Protein-bound SRB was solubilized with 150 μl 10 mM Tris-HCl, pH 10. Optical densities were measured at 560 nm on a microplate reader (Bio-rad, Hercules, CA). Proliferation is expressed as fold increase, normalized for the protein content of control cells 1 day after plating.

Alternatively, cell proliferation was measured using the xCELLigence Real-Time Cell Analyzer system (ACEA Biosciences, San Diego, CA). Cells were plated in duplicate at a density of 1000 cells/well on ACEA E16 view plates. The next day, EGCG or vehicle was added to the wells. Doubling times were calculated over 48 h, using dedicated ACEA software.

### GLUD1/2 and IDH enzymatic assays

IDH1 was expressed as glutathione S-transferase (GST)-fusion protein in pDEST15 and purified on glutathione beads (GE Healthcare, Chicago, IL) as described [34]. Purified bovine GLUD1/2 was purchased from Serva (Heidelberg, Germany). Enzyme reactions were initiated by adding 4 μg IDH1 enzyme to a mixture of 100 μM NADP+, 2 mM MgCl_2_, 0.5 mM isocitrate, and 100 mM Tris-HCl (pH 7.4). GLUD1/2 activity was measured in reactions containing 0.1 U bovine GLUD1/2 enzyme, 500 μM NAD^+^, 10 mM glutamate, and 2 mM ADP in phosphate buffer (pH 8.0). Stoichiometric production of NADPH and NADH was measured by real-time monitoring of NADPH or NADH absorbance at 340 nm with 20 s intervals on an Omega Fluostar (BMG Labtech, Ortneberg, Germany).

### Colony-forming assays after ionizing radiation (IR)

Cells, cultured with or without AGI-5198, were seeded in 6-well plates (30–5000 cells/well) and left to adhere overnight. Cells were treated with 0, 20, 50, or 100 μM EGCG for 24 h and irradiated with 0, 2, or 4 Gy (IR, 3.1 Gy/min; XRAD 320 ix; Precision XRT; N. Brandford, CT, USA). After 72 h, the medium was refreshed and cells were cultured for another 7 days (without EGCG) and fixated with 70% ethanol (10 min, 4 °C). After drying at 60 °C, colonies were stained with 0.5% (*w*/*v*) crystal violet (Merck) in distilled water. Colonies consisting of 50 cells or more were considered to be derived from cells surviving radiotherapy and were manually counted. The effect of EGCG on radiotherapy-induced cell death was expressed as surviving fraction, normalized to plating efficiency.

### DNA-double strand break (DSB) detection

Cells (cultured with or without AGI-5198) were plated at a density of 300,000 cells/well in 6-well plates and left to adhere overnight. After 24 h incubation with EGCG (0, 50, or 100 μM), cells were irradiated with 0, 2, or 4 Gy. After 30 min, cytosolic extracts were prepared in 1× RIPA buffer (Cell Signaling Technologies) containing 1 mM phenylmethylsulfonyl fluoride (PMSF). Cell extracts were sonicated to release nuclear proteins. Protein samples (25 μg) were electrophoresed on 10% SDS-PAGE gels and electroblotted onto nitrocellulose (GE Healthcare). Blots were stained with anti-γH2AX antibody (Ser139; #2577; Cell Signaling Technologies) and anti-γ-tubulin (C20) (Santa Cruz Biotechnology, Dallas, TX, sc-7396), followed by appropriate secondary antibodies labeled with IRDye680 or IRDye800 (ThermoFisher). Signals were visualized and quantified using the Odyssey system (Li-COR, Lincoln, NE).

### Statistical analysis

Statistical analyses were performed in GraphPad Prism v5.03 (GraphPad Software, LaJolla, CA). The difference in mean values between various groups was assessed using an unpaired Student’s *t* test, unless mentioned otherwise. *P* values are marked as follows: < 0.05 (*); < 0.01 (**); < 0.001 (***), < 0.0001 (****). Differences were considered statistically significant when *P* values were < 0.05.

## Results

### Glutamine and glutamate are carbon donors for *D-*2-HG production

To find support for the hypothesis that glutaminolysis and/or glutamatolysis is a rescue mechanism for *IDH*^*mut*^ cancers, we used the HCT116 cell line and its isogenic knock-in variant HCT116-*IDH1*^*wt/R132H*^. The balanced expression of both alleles makes this variant more representative for clinical cancers than overexpression models [35]. We used [1-^13^C]-labeled glutamine (Gln*) and glutamate (Glu*) at similar concentrations to trace the routing of carbons from Gln and Glu to *D-*2-HG. Labeled carbon from Glu or Gln that enters the TCA cycle is lost as carbon dioxide by oxidative decarboxylation of α-KG (see Fig. 1a).

^13^C-NMR spectra of extracts of *IDH1*^*mut*^ cells, cultured for 20 h in Gln*-containing DMEM, showed ^13^C resonances for Glu and *D-*2-HG, demonstrating significant carbon fluxes from Gln to Glu and to *D-*2-HG (see blue graph in upper panel of Fig. 1b for a representative NMR spectrum). In similar experiments with Glu* instead of Gln* in the medium, we observed ^13^C resonances for Gln and *D-*2-HG, next to that for Glu, demonstrating carbon flux from Glu to Gln and to *D-*2-HG (see red spectrum in the upper panel of Fig. 1b, c). Whereas cells take up less Glu* than Gln*, the ratio *D-*2-HG*/Glu* (0.36, *n* = 2) was higher than that of *D-*2-HG*/Gln* in the Gln* incubation experiments (0.08, *n* = 6; see Fig. 1c). Since *D-*2-HG was not detected in parental HCT116 cells (see lower panel of Fig. 1b), these cells were not used for further ^13^C experiments.

Relative contributions of Glu, Gln, and Glc carbons to the total pool of *D-*2-HG were assessed with LC-MS of extracts of cells, cultured 20 h in medium with Glu* or Gln*, together with Glc* ([1,6-^13^C_2_]-glucose). Experiments with Gln* + Glc* in the medium revealed that 64 ± 8.5% of *D-*2-HG was derived from Gln* whereas 22 ± 3.8% was derived from Glc* (*n* = 5; see Fig. 1d, medium formulation I). When cells were cultured in Glu* + Glc*-containing DMEM in the absence of Gln, the total amount of *D-*2-HG decreased, with 53 ± 3.1% of carbons originating from Glu* and 31 ± 1.8% from Glc* (*n* = 3; Fig. 1d, formulation II). Only small amounts of non-labeled *D-*2-HG were detected when cells were cultured in medium formulations I and II. When cultured in DMEM with Glu* and non-labeled Gln and Glc, the total amount of *D-*2-HG was highest (Fig. 1d, formulation III). Of all intracellular *D-*2-HG, 41 ± 0.2% was derived from Glu* (*n* = 3). The latter experiment showed that direct glutamate contribution is substantial even in the presence of glutamine and that the availability of Gln is a prerequisite for increased *D-*2-HG production.

### EGCG inhibits proliferation of *IDH*^*mut*^ cells more effectively than proliferation of *IDH*^*wt*^ cells

**Fig. 2:**
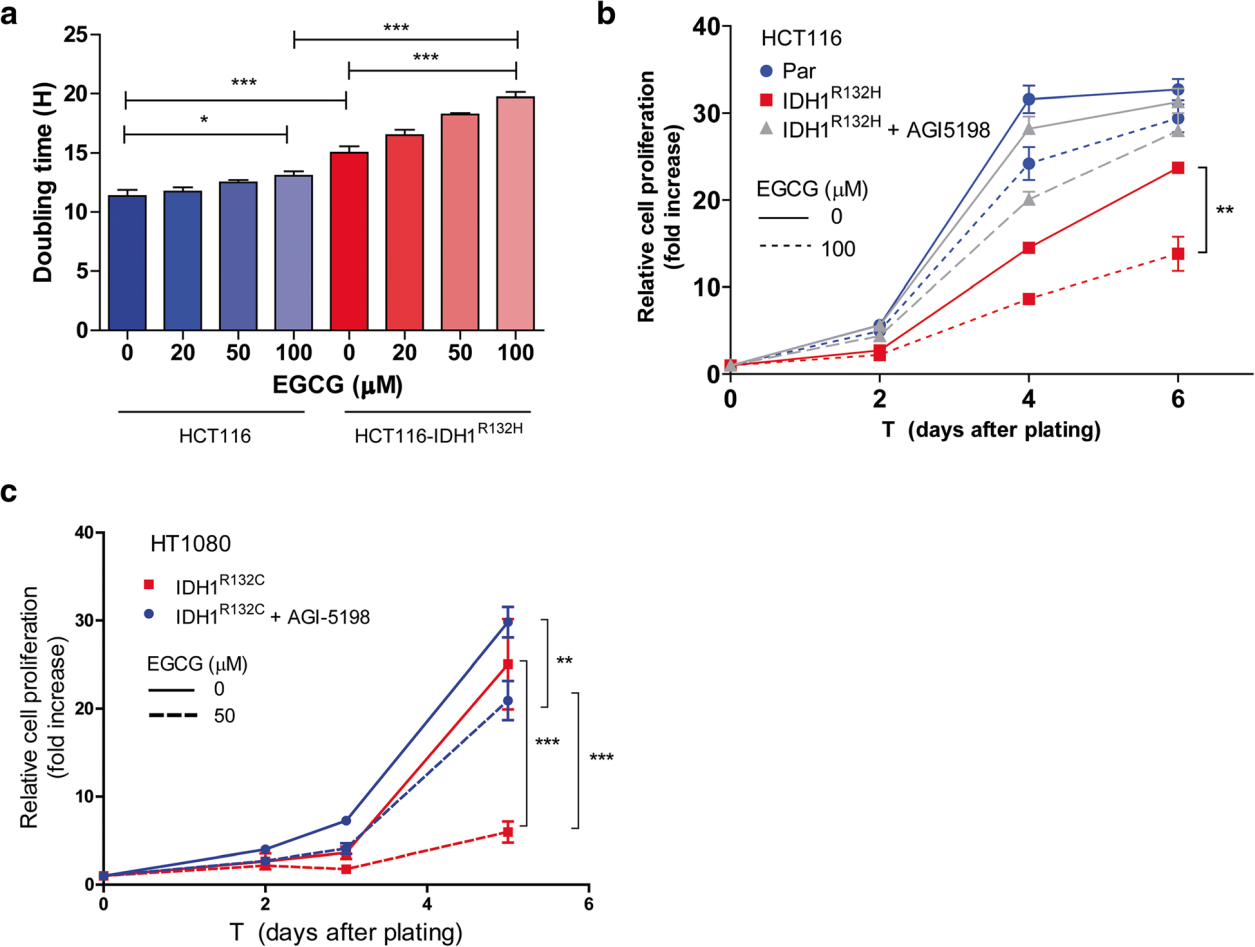
Proliferation assays of the isogenic cell line pair HCT116 and HCT116-*IDH1*^*wt/R132H*^ with EGCG. **a** Doubling times of cells as measured on the xCELLigence. Doubling times for HCT116-*IDH1*^*wt/R132H*^ (red bars) were significantly higher compared to its wild-type counterpart (blue bars). Supplementation of EGCG to the culture medium increased doubling times in both cell lines, but this effect was more significant in HCT116-*IDH1*^*wt/R132H*^*.* **b** SRB assay of HCT116 cells cultured with EGCG (dotted lines) or vehicle (solid lines). HCT116-*IDH1*^*wt/R132H*^ (red lines) cells had slower proliferation rates as compared to HCT116 (blue lines)*.* Addition of AGI-5198 (gray lines) rescued the proliferation rates of *IDH1*^*R132H*^ cells to HCT116 baseline levels. EGCG inhibited proliferation of HCT116-*IDH1*^*wt/R132H*^ more than HCT116, and this inhibitory effect was abolished by AGI-5198. Similar results were obtained for HT10180 cells (**c**)

Our finding that the glutamine-glutamate pathway is an important carbon donor for *D-*2-HG via α-KG suggests that blocking this pathway not only decreases α-KG availability and *D-*2-HG production, but also increases oxidative stress [21]. In line with this hypothesis, inhibiting GLUD1/2 by EGCG dose-dependently reduced growth rates of HCT116-*IDH1*^*wt/R132H*^ cells more than of parental HCT116 cells (Fig. 2a). Because the IDH1^mut^ inhibitor AGI-5198 prevents *D-*2-HG production and NADPH oxidation [21], it is expected to annihilate the metabolic stress that is caused by the *IDH* mutation, thereby reducing the effects of EGCG. Indeed, AGI-5198 treatment antagonized the inhibitory effect of EGCG to the level that was observed for parental HCT116 cells (Fig. 2b). This finding was supported in experiments with HT1080 cells, in which AGI-5198 treatment resulted in significantly increased proliferation rates, while decreasing sensitivity to EGCG (Fig. 2c).

### EGCG inhibits GLUD1/2 and IDH1 activity

**Fig. 3:**
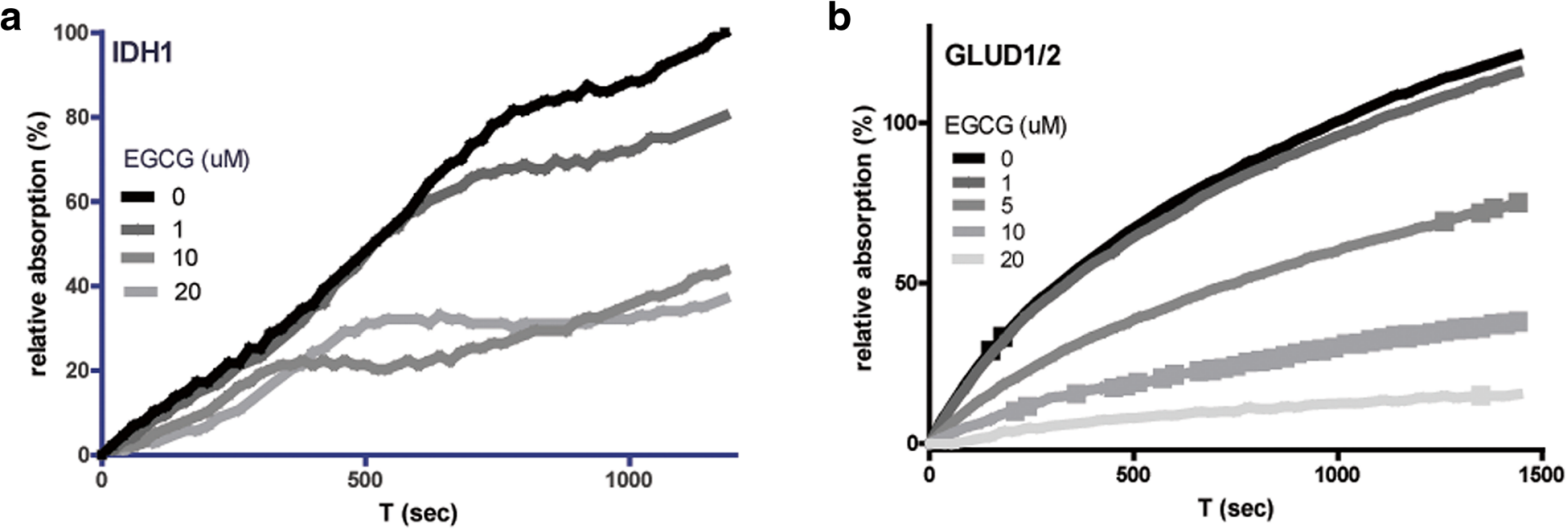
EGCG inhibits the activity of IDH1 and GLUD1/2. IDH1 (**a**) and GLUD1/2 (**b**) activities were inhibited by EGCG, but this effect was much more pronounced in GLUD1/2. Activity was determined by measuring NADP(H)-generated absorption at 340 nm. Activity was corrected for absorption measured with cofactors, without the addition of enzyme

EGCG is an inhibitor of GLUD but also of other NADP^+^-dependent enzymes [36], which could contribute to the reduction in growth rates of HCT116 and HT1080 cells. We therefore tested the effects of EGCG on enzymatic activities of wild-type IDH1 and GLUD1/2 in biochemical assays, quantifying NAD(P) H production by 340 nm absorption. These experiments revealed dose-dependent inhibition of both GLUD1/2 and of IDH1 activity (Fig. 3).

### EGCG reduces *D-*2-HG production in HCT116-*IDH1*^*wt/R132H*^

**Fig. 4:**
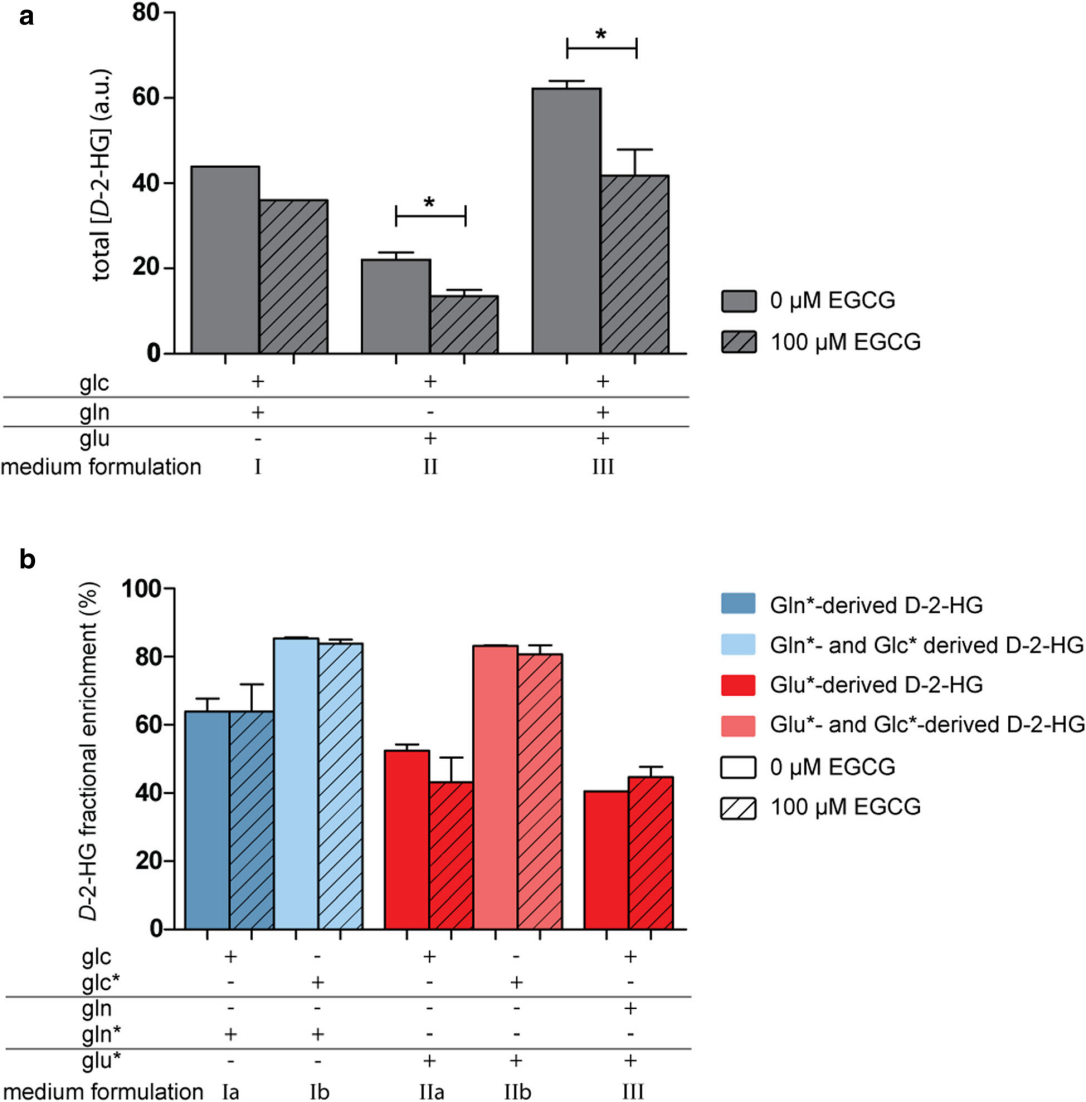
LC-MS analysis of *D-*2-HG pools after treatment with EGCG. HCT116-*IDH1*^*wt/R132H*^ cells were incubated in DMEM with 10% FCS, supplemented with glucose, glutamine, and/or glutamate as indicated in the presence or absence of EGCG (shaded vs. non-shaded bars respectively). **a** Total pools of *D-*2-HG after incubation in medium containing Glc and Gln (I), Glc and Glu (II), or Glc, Gln, and Glu (III). Total *D-*2-HG levels decreased when cultured with EGCG. Fractional enrichments (FE) of *D-*2-HG are displayed in **b**. Blue bars display the fractions of *D-*2-HG that were derived from Gln* (dark blue) and both Gln* and Glc* (light blue) after incubation in DMEM with Glc + Gln* (formulation Ia) and Glc* + Gln* (formulation Ib) respectively. Red bars display the fractions of *D-*2-HG that were derived from Glu* (dark red) and both Glu* and Glc* (light red) after incubation in DMEM with Glc + Glu* (formulation IIa) and Glc* + Glu* (formulation IIb) respectively, and the fraction of *D-*2-HG derived from Glu* (dark red) after incubation in DMEM with Glc, Gln, and Glu* (formulation III)

The effect of EGCG on IDH1 and GLUD1/2 activity predicts that EGCG inhibits the formation of α-KG and *D-*2-HG in *IDH1*^*mut*^ cells. To test this hypothesis, we analyzed the effects of EGCG on *D-*2-HG production in HCT116-*IDH1*^*wt/R132H*^ cells using LC-MS. Total amounts of *D-*2-HG were decreased in cells treated with EGCG (Fig. 4a). This alteration was most apparent in cells incubated in DMEM containing Glc, Glu, and Gln. EGCG treatment decreased carbon flux from Glu* to *D-*2-HG, although this difference was not statistically significant (Fig. 4b). Because EGCG binds to serum albumin [37], possibly diminishing cellular uptake, we repeated the LC-MS experiment, but now cultured cells in serum-free medium. Under these conditions, EGCG treatment resulted in a significant reduction of ^13^C- flux from *Glu to *D-*2-HG (Additional file 1: Figure S1).

### EGCG increases sensitivity of HCT116-*IDH1*^*wt/R132H*^ cells to radiotherapy

**Fig. 5:**
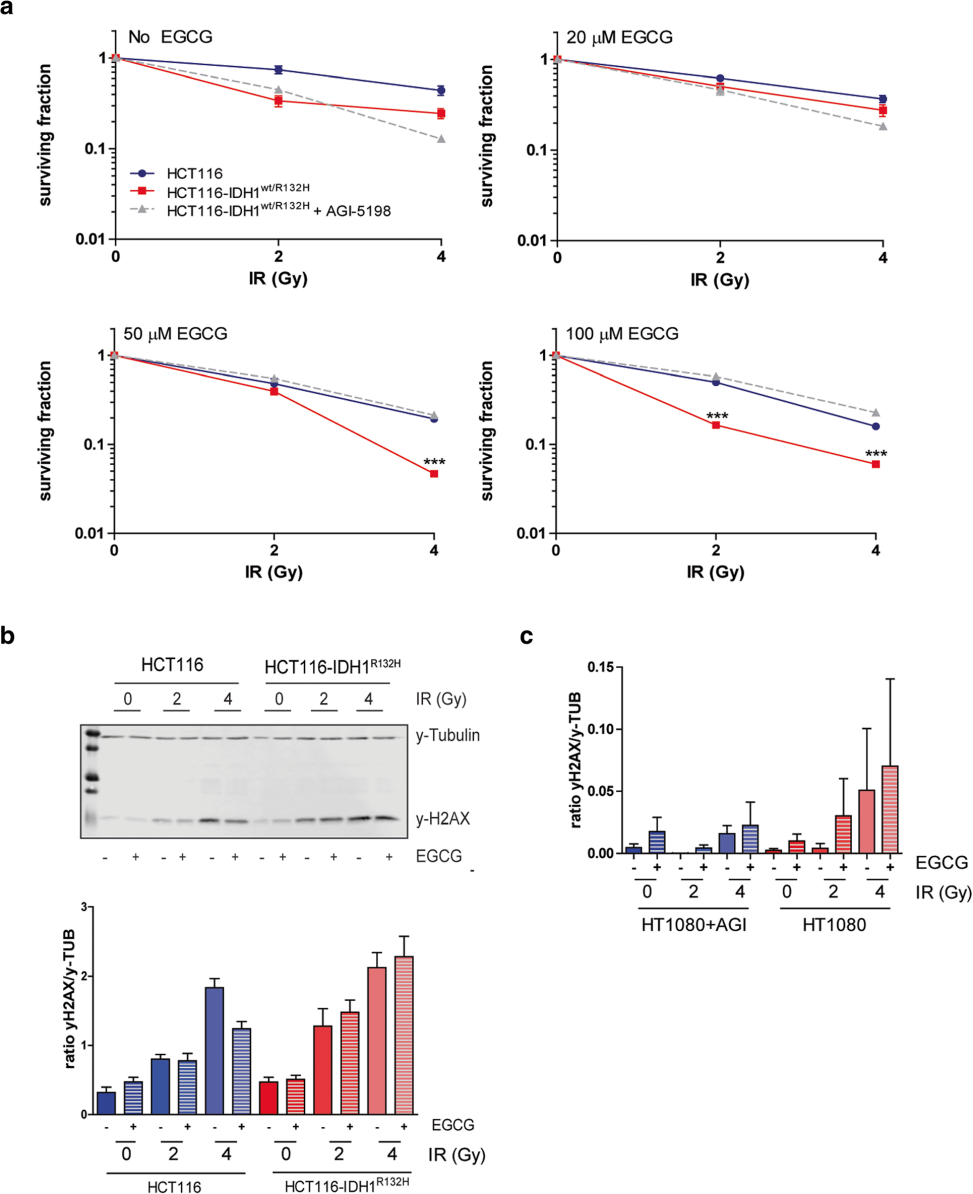
EGCG increases radiosensitivity in HCT116-*IDH1*^*wt/R132H*^ cells. **a** Colony formation assay of HCT116-*IDH1*^*wt/R132H*^ cells shows increased sensitivity to IR after treatment with EGCG compared to in HCT116. Surviving fractions were 5% (± 0.5%) vs. 16% (± 2%) for *IDH1*^*wt/R132H*^ and *IDH1*^*wt*^ cells, respectively, when cultured with 100 μM EGCG and irradiated with 4Gy. **b** Western blot quantification of phosphorylated H2AX foci in HCT116-*IDH1*^*wt/R132H*^ and HCT116 cells, cultured with or without 100 μM EGCG and irradiated at 2 or 4 Gy. Endogenous H2AX-phosphorylation was higher in HCT116-*IDH1*^*wt/R132H*^ cells than in HCT116 cells. Thirty minutes after IR, γH2AX levels further increased to higher levels in HCT116-*IDH1*^*wt/R132H*^ than in HCT116 cells. The same trend could be observed in HT1080 (+ AGI-5198) cells (**c**)

Because EGCG inhibits GLUD1/2 and IDH1 activity and thus NADH and NADPH production, it is expected to increase oxidative stress and sensitize cells for ionizing radiation. Colony formation assays showed that EGCG increased the sensitivity to IR of both cell lines, although at low doses (20 μM) EGCG appeared to protect cells against radiotherapy (Fig. 5a, Table 1). At higher doses of EGCG, HCT116-*IDH1*^*wt/R132H*^ cells were significantly more sensitive to IR than HCT116 cells. Inhibition of IDH1^R132H^ activity with AGI-5198 resulted in decreased radiosensitivity of EGCG-treated HCT116-*IDH1*^*wt/R132H*^ cells (dotted gray lines).

**Table 1:** Cell survival of irradiated cells. Surviving fractions are presented as percentage surviving colonies (± standard deviation), normalized to the non-irradiated control per EGCG treatment condition. Colonies > 50 cells were considered to be derived from cells that survived radiotherapy. Surviving fractions were normalized to plating efficiency. The surviving fractions are also visualized in Fig. 5a

	EGCG (μM)			
IR (Gy)	0	20	50	100
HCT116				
0	100 (± 0)	100 (± 0)	100 (± 0)	100 (± 0)
2	74 (± 12)	62 (± 3)	48 (± 11)	50 (± 3)
4	44 (± 9)	37 (± 6)	19 (± 1)	16 (± 2)
HCT116-IDH1^wt/R132H^				
0	100 (± 0)	100 (± 0)	100 (± 0)	100 (± 0)
2	33 (± 8)	50 (± 3)	39 (± 6)	16 (± 0.5)
4	24 (± 5)	28 (± 7)	5 (± 0.5)	5 (± 0.5)
HCT116-IDH1^wt/R132H^ + AGI-5198				
0	100 (± 0)	100 (± 0)	100 (± 0)	100 (± 0)
2	44 (± 11)	46 (± 9)	55 (± 3)	57 (± 17)
4	13 (± 4)	18 (± 2)	21 (± 2)	22 (± 6)

In addition to colony assays, we also measured γH2AX to assess IR sensitivity. Phosphorylation of histone H2AX is a rapid response to DNA-DSBs [38]. To test the direct IR-induced DNA damage, we determined levels of γH2AX in HCT116, HCT116-*IDH*^*wt/R132H*^, and HT1080 cells with and without AGI-5198, 30 min after irradiation with 0, 2, or 4 Gy. Endogenous levels of γH2AX were higher in HCT116-*IDH1*^*wt/R132H*^ than in HCT116 cells (Fig. 5b) as reported before [21]. Higher irradiation doses resulted in increased levels of γH2AX, and there was a slight trend towards increased γH2AX levels after EGCG treatment of HCT116-*IDH*^*wt/R132H*^, but not HCT116, cells. A similar trend was observed in HT1080 cells although differences did not reach statistical significance (Fig. 5c). Of note, treatment with AGI-5198 reduced the amount of γH2AX as was shown before [21].

## Discussion

Since the discovery of the frequent occurrence of *IDH1* mutations in various types of cancer, research has mainly focused on the oncogenic effects of *D-*2-HG, the product of the mutant enzyme [39]. Recognizing the oncogenic role of *D-*2-HG, IDH1^mut^- and IDH2^mut^-specific drugs have been developed that inhibit the activity of the mutant enzymes [19]. We recently showed that these inhibitors not only inhibit the production of *D-*2-HG, but also prevent NADPH oxidation [21]. This results in normalization of the redox status of the cell and thus decreased sensitivity to IR, which may imply that these inhibitors are contra-indicated for combination treatment with IR. In glioma, mutations in *IDH1* are considered ancestral, driving gliomagenesis but not necessarily glioma progression [17]. In the initial stages of neoplastic transformation, *D-*2-HG affects the epigenome through inhibition of α-KG-dependent DNA-and histone-demethylases [40]. However, to overcome metabolic stress induced by IDH mutations [41], during the progression of the disease, *IDH*^*mut*^ cells need to adopt rescue mechanisms that supply the cells with the required basic level of α-KG to remain viable. The existence of such rescue pathways could explain the finding that α-KG levels are only slightly reduced in the IDH1^wt/R132H^-mutant glioma model E478 [32], a finding that was also made in other studies [13, 42]. Identification of these pathways potentially allows the rational selection of metabolic inhibitors for therapeutic applications.

Previous in vitro and in vivo studies reported that *IDH*^*mut*^ cells rely on glutaminolysis for anaplerosis of glutamate and production of reduced glutathione, an important scavenger of reactive oxygen species [26, 29, 43, 44]. We recently postulated that metabolic rescue mechanisms involve direct import and anaplerotic consumption of glutamate and lactate in *IDH*^*mut*^, but not *IDH*^*wt*^ gliomas based on transcriptome and MR spectroscopy experiments [16, 25, 30]. Glutamate import is regulated via excitatory amino acid transporters (EAAT) which are expressed at high levels in IDH^mut^-glioma cells [25] but at low levels in HCT116 cells [45]. In the present work, we provide direct evidence using ^13^C-tracing that glutamine and glutamate are carbon sources for *D-*2-HG production, even in HCT116-*IDH1*^*mut*^ cells. In this study, we used in vitro glutamate concentrations of 4 mM, which is higher than what is observed in plasma in vivo and higher than in the extracellular space in normal brain [46]. An open question is what the levels of extracellular glutamate concentrations are in brain tumors. As shown in Fig. 1, the presence of glutamine in culture medium increased the intracellular pool of *D-*2-HG in HCT116-*IDH1*^*wt/R132H*^ cells. In medium with *Glu in the absence of Gln, less *D*-2-HG was produced but the contribution of *Glu-derived carbon in *D*-2-HG was relatively high. We postulate that the lower total *D-*2-HG pools are attributed to lower uptake of glutamate relative to glutamine by HCT116-*IDH1*^*wt/R132H*^ cells.

Most research on *IDH*^*mut*^ cancers is currently performed on cell lines that overexpress IDH1^mut^ [47, 48]. Recent evidence suggests that the *IDH* mutation may be one of the initial mutations that occur in glioma [49, 50]. Establishing tumor models that carry the endogenous *IDH1*^*wt/R132H*^ mutation is difficult [51, 52], and overexpression models are not necessarily representative for the heterozygous mutation that occurs in these cancers [53]. Therefore, we here used the heterozygous HCT116-*IDH1*^*wt/R132H*^ knock-in cell line, one of the few in vitro models carrying a heterozygous *IDH1*^*wt/R132H*^ mutation. The use of the isogenic cell line pair (HCT116-*IDH1*^*wt/R132H*^ and HCT116) allowed us to link the *IDH1* mutation to sensitivity to GLUD inhibition. Our results may also have relevance to other *IDH1*^*mut*^ cancer types, as *IDH* mutations are also found in colorectal cancers, although at low frequency [17, 54]. Even though the importance of glutamate and glutamine as an anaplerotic fuel might differ between cancer types due to the nature of cells, inhibition of the processing pathway downstream of glutamine will hamper the cancer cells either way.

Previous studies have shown that the glutaminase (GLS) inhibitor BPTES inhibits proliferation of *IDH*^*mut*^ cells [48]. The potential of cells to bypass GLS activity by directly using glutamate instead of glutamine is a possible explanation for the relatively small inhibitory effect of BPTES in that study. Therefore, inhibiting glutamatolysis at the level of GLUD1/2 may be a more effective strategy. EGCG affects the production of *D-*2-HG, among others, by diminishing the supply of α-KG by inhibition of GLUD1/2 but likely also by inhibition of the IDH1^wt^ subunit, and simultaneously deteriorates the redox status by inhibiting NAD(P) H production. In this way, EGCG has clear benefits over inhibitors that only inhibit GLS or mutant IDH activity directly. Glu-derived *D-*2-HG production was inhibited more by EGCG than Gln-derived *D*-2-HG. We explain this by the fact that GLUD has both a mitochondrial and cytosolic isoform, while GLS is mitochondrial. As EGCG cannot enter the mitochondrial matrix [55] Gln may lead to the production of mitochondrial glutamate and α-KG that may be processed in the TCA cycle instead of being converted in the cytosol *D*-2-HG.

Cell growth was reduced by EGCG in both HCT116 and HCT116-*IDH1*^*wt/R132H*^ cell lines. We attribute this to the fact that EGCG inhibits multiple NADP^+^-dependent enzymes including IDH1^wt^ [36]. We hypothesize that the significantly larger effect of EGCG on HCT116-*IDH1*^*wt/R132H*^ cells is caused by a higher GLUD dependency compared to HCT116 cells. However, it should be realized that EGCG is a pleiotropic compound, and activities other than GLUD and IDH inhibition also may have played a role in the observed effects.

A direct relationship between EGCG and increased oxidative stress was established by its sensitizing effect on radiotherapy, which was significantly higher in HCT116-*IDH1*^*wt/R132H*^ cells, as demonstrated by decreased survival in colony forming assays and as suggested by increased DNA-DSB after IR at high doses of EGCG. Interestingly, a recent report described that *D-*2-HG in IDH1^mut^ cancers inhibits the activity of the α-KG-dependent DNA repair enzyme alkB homolog (ALKBH) [56]. This may provide an additional explanation for increased baseline sensitivity of *IDH1*^*mut*^ cancers to radiation therapy and alkylating chemotherapy. The decreased IR sensitivity of HCT116-*IDH1*^*wt/R132H*^ treated with AGI-5198 is in agreement with this hypothesis.

EGCG has several other effects next to the inhibition of GLUD. It can inhibit histone and DNA demethylases, reactivate tumor suppressor genes [57–59], and inhibit fatty acid synthase [60] and glucose-6-phosphate dehydrogenase, the rate-limiting enzyme in the pentose phosphate pathway and a provider of NADPH [61]. Furthermore, EGCG has been reported to inhibit epidermal growth factor (EGF)-induced activation of the EGF-receptor (EGFR) [62], a frequently encountered aberrant oncogenic pathway in glioblastoma and many other cancers. The combination of these effects provides a solid rationale to test EGCG as an adjuvant treatment to radiotherapy, and possibly chemotherapy, also in *IDH*^*wt*^ cancers. However, our data also show that the effects of EGCG may be dose-dependent, having a radioprotective effect on HCT116-*IDH1*^*wt/R132H*^ cells at low doses. This may be due to both its anti-oxidative and pro-oxidative effects [63] and warrants extra investigation.

One problem that requires attention is the bioavailability and stability of EGCG in vivo [55, 64, 65]. Anticancer effects of EGCG can be diminished due to oxidation of the compound [66], resulting in low circulating doses after oral administration [67, 68]. Furthermore, EGCG in the circulation is mostly albumin-associated, increasing stability but decreasing bioavailability [37]. Methods to increase bioavailability via nanomedicine and controlled delivery are currently being explored [69, 70].

## Conclusions

HCT116-*IDH1*^*wt/R132H*^ cells use glutamine and glutamate as direct sources for α-KG anaplerosis and *D-*2-HG production. EGCG, a derivative of green tea, inhibits glutamate processing at the level of GLUD1/2, reduces proliferation rates, decreases production of *D-*2-HG, and increases sensitivity to radiotherapy in *IDH1*^*wt/R132H*^ cells. Additional studies are required to test these concepts in vivo and to investigate effective ways of delivering EGCG to the brain.

## Additional file

Additional file 1: **Figure S1.** LC-MS analysis of *D-*2-HG pools in cells cultured in serum-free medium. HCT116-*IDH1*^*wt/R132H*^ cells were incubated in DMEM without FCS and with and without EGCG (shaded vs. non-shaded bars respectively). Blue bars display the fractions of *D-*2-HG that were derived from Gln* (dark blue) and both Gln* and Glc* (light blue) after incubation in DMEM supplemented with Glc + Gln* (formulation Ia) and Glc* + Gln* (formulation Ib) respectively. Red bars display the fractions of *D-*2-HG that were derived from Glu* (dark red) and both Glu* and Glc* (light red) after incubation in DMEM with Glc + Glu* (formulation IIa) and Glc* + Glu* (formulation IIb) respectively, and the fraction of *D-*2-HG that was derived from Glu* (dark red) after incubation in DMEM with Glc, Gln and Glu* (formulation III). Fractional enrichments of *D-*2-HG decreased significantly when incubated with EGCG. The glucose fraction can be obtained by calculating the difference Ib-Ia and IIb-IIa. Supplemented metabolite concentrations were always the same: 5.5 mM glucose and 4 mM glutamine and/or 4 mM glutamate. (TIF 1584 kb)

## Abbreviations

ALKBH: AlkB homolog
BSA: Bovine serum albumin
*D-*2-HG: *D*-2-hydroxyglutarate
*D-*2-HG*: [1-^13^C]- *D*-2-hydroxyglutarate
D_2_O: Deuterated water
DAPI: 4′,6-diamidino-2-phenylindole
DSB: Double-strand break
EGCG: Epigallocatechin-3-gallate
EGF: Epidermal growth factor
EGFR: Epidermal growth factor receptor
FCS: Fetal calf serum
Glc: Glucose
Glc*: [1,6-^13^C_2_]-glucose
Gln: Glutamine
Gln*: [1-^13^C]-glutamine
GLS: Glutaminase
Glu: Glutamate
Glu*: [1-^13^C]-glutamate
GLUD: Glutamate dehydrogenase
H2AX: H2A histone family member X
ICT: Isocitrate
IDH: Isocitrate dehydrogenase
IR: Ionizing radiation
LC-MS: Liquid chromatography mass spectroscopy
NMR: Nuclear magnetic resonance
NS: Number of averages
PARP: Poly-ADP ribose polymerase
SRB: Sulforhodamine B
TCA: Tricarboxylic acid
TET: Ten-eleven translocation
TR: Repetition time
α-KG: α-ketoglutarate
γH2AX: Phosphorylated H2AX
