# Supplementary figures and images for: Isocitrate dehydrogenase 1-mutated cancers are sensitive to the green tea polyphenol epigallocatechin-3-gallate
Source: Cancer Metab. 2019 May 20;7:4. doi: 10.1186/s40170-019-0198-7 (PMC6526618; doi:10.1186/s40170-019-0198-7)

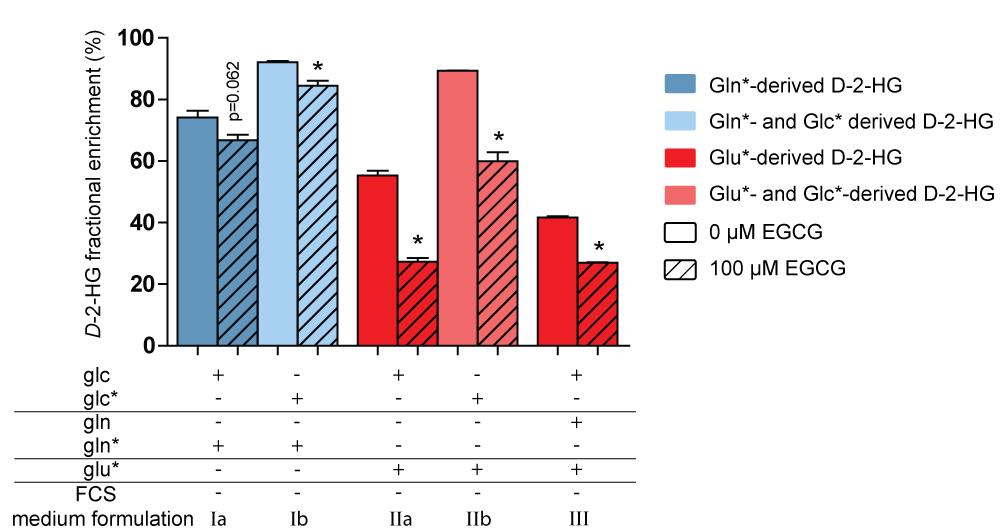

Supplement: Supplementary file 1 — Figure S1. LC-MS analysis of D-2-HG pools in cells cultured in serum-free medium. HCT116-IDH1wt/R132H cells were incubated in DMEM without FCS and with and without EGCG (shaded vs. non-shaded bars respectively). Blue bars display the fractions of D-2-HG that were derived from Gln* (dark blue) and both Gln* and Glc* (light blue) after incubation in DMEM supplemented with Glc + Gln* (formulation Ia) and Glc* + Gln* (formulation Ib) respectively. Red bars display the fractions of D-2-HG that were derived from Glu* (dark red) and both Glu* and Glc* (light red) after incubation in DMEM with Glc + Glu* (formulation IIa) and Glc* + Glu* (formulation IIb) respectively, and the fraction of D-2-HG that was derived from Glu* (dark red) after incubation in DMEM with Glc, Gln and Glu* (formulation III). Fractional enrichments of D-2-HG decreased significantly when incubated with EGCG. The glucose fraction can be obtained by calculating the difference Ib-Ia and IIb-IIa. Supplemented metabolite concentrations were always the same: 5.5 mM glucose and 4 mM glutamine and/or 4 mM glutamate. (TIF 1584 kb) [file 40170_2019_198_MOESM1_ESM.tif]
